# Barrett's Esophagus: Where Do We Stand?

**DOI:** 10.4103/1319-3767.45046

**Published:** 2009-01

**Authors:** Majid A. Al Madi

**Affiliations:** Department of Gastroenterology, McGill University, Montreal, Canada

**Keywords:** Ablation, Barrett's esophagus, cancer, chemoprevention, dysplasia, esophagus, guidelines, imaging, screening, surveillance

## Abstract

Barrett's esophagus (BE) is a precursor for esophageal adenocarcinoma, which has an increased incidence rate over the last few decades. Its importance stems from the poor five-year survival of esophageal adenocarcinoma and current data that suggest a survival benefit when surveillance programs are implemented. In this review, we will cover the pathophysiology and natural history of BE and the different endoscopic findings. The prevalence of BE in different geographic areas and the incidence of high-grade dysplasia and adenocarcinoma in this patient population is reviewed. Recent recommendation for screening and surveillance of BE has been covered in this review as well as the efficacy of nonconventional imaging modalities and endoscopic ablation therapies.

Esophageal cancer incidence has rapidly accelerated over the last few decades with incidence rates in western countries surpassing other cancers like breast and prostate cancer.[1] Special attention is being paid to Barrett's esophagus (BE) as it has been recognized as a precursor for esophageal adenocarcinoma (EAC). The latest guidelines from the American College of Gastroenterology have redefined BE as “a change in the distal esophageal epithelium of any length that can be recognized as columnar type mucosa at endoscopy and is confirmed to have intestinal metaplasia by biopsy of the tubular esophagus”;[2] whereas the previous definition cited the “displacement of the squamocolumnar junction proximal to the gastroesophageal junction and endoscopy with multiple systematic biopsies needed to establish the diagnosis of Barrett's esophagus.”[3] Intestinal metaplasia (IM) is clearly cited as a prerequisite to make the diagnosis in the more recent definition of BE. In addition, surveillance programs have been implicated for this patient population to detect high-grade dysplasia (HGD) or early adenocarcinoma with the hope of improving survival rates.

## PATHOPHYSIOLOGY

The development of BE occurs in response to oxidative damage and inflammation inflicted on the mucosa from contact with the gastric contents. The end result of this cascade is metaplasia of the normal squamous lining of the esophagus to intestinal-type columnar epithelium that has the potential to develop EAC. There is definite genetic predisposition to the development of BE, as it has been demonstrated that there is an increase in the risk of EAC in patients with GERD or BE carrying the genotypes for epidermal growth factor (EGF) A61G G/G, cyclooxygenase 2 (COX2) 8473 C.[4,5] Also an animal study suggested that multipotential progenitor cells of bone marrow origin may play a role in metaplasia in BE.[6] Multiple inflammatory mediators have become a target for researchers in the hope of decreasing the progression to adenocarcinoma, especially after studies demonstrated that superoxide dismutase and COX2 inhibitors decreased the progression of BE and adenocarcinoma in animal models of BE.[7–9] Of these mediators, secretory phospholipase A,[2] eicosanoid, COX2, prostaglandin E2 and leukotriene B4[10–12] have been targeted; the use of the n3 fatty acid eicosapentaenoic acid (EPA) has been shown to decrease COX2 protein concentration.[10] Also, aspirin in combination with a PPI was demonstrated to decrease the concentration of prostaglandin E2.(12) A meta-analysis demonstrated a protective effect of aspirin and nonsteroidal antiinflammatory drugs (NSAIDs) from esophageal cancer,[13] whereas the use of COX2 inhibitors did not alter the natural history of BE in a number of randomized controlled trials (RCTs) including the Chemoprevention for BE Trial (CBET).[14,15] In addition, multiple intracellular signaling proteins that regulate EAC proliferation and apoptosis have been identified and targeted recently such as extracellular signal-regulated kinase (ERK) and protein kinase B (Akt) using statins[16,17] as well as iron import proteins.[18]

## NATURAL HISTORY

The prevalence of BE in patients with gastroesophageal reflux disease (GERD) has been reported to be about 10–20% in western countries[19] and about 0.2–5% in Asia.[20,21] The prevalence of BE in patients undergoing upper endoscopic procedures for any reason was found to be 2.1%.[19,22] Published data on the natural history of BE has been variable, with the prevalence of EAC in patients with BE in the range of 5%.[23] It has been noted that there is a difference in the prevalence of BE among different ethnicities and geographical areas, with a high prevalence in the western hemisphere and being less in blacks, Hispanics, and Asians.[24–26] A recent meta-analysis by Yousef *et al*[27] estimated the cancer risk in patients with BE to be 6.1/1000 person-years, and when early cancer and high-grade dysplasia (HGD) were excluded to be 4.1/1000 person-years and that men progressed to cancer at twice the rate when compared with women. A second meta-analysis showed that in patients with BE and HGD, the rate of developing adenocarcinoma was 6/100 person-years[28] and that rate did not differ on the basis of geographical areas.[29] Dietary habits have been associated with the risk of the development of BE, as the consumption of a western diet (rich in fast foods and meat) had an adverse effect on the development of BE, and an inverse relationship was found between BE and a diet rich in fruits, vegetables, and nonfried fish.[30] Other risk factors identified for the development of BE are male gender, age more than 40 years, heartburn more than once per week, and long duration of symptoms (>13 years).[21,31–36] A recent meta-analysis addressed the issue of increased adiposity as a risk factor for developing BE and found that it had an indirect relationship through an increase in GERD rather than a direct effect.[37] Other causes of mortality apart from adenocarcinoma in patients with BE were bronchopneumonia and ischemic heart disease.[38,39]

Although Helicobacter pylori does not have any influence on esophageal acid reflux or on symptoms in patients with BE,[40] a meta-analysis demonstrated an inverse relationship between *H. pylori* infection and BE and esophageal adenocarcinoma, suggesting a protective effect of this infection.[41,42] This can explain to a degree the low prevalence of BE in areas with a high prevalence of *H. pylori* infection.

## ENDOSCOPIC FINDINGS

BE is usually easily recognized with its typical salmon-colored mucosa that contrasts with the normal pearly white esophageal squamous mucosa. Also, BE is not appreciated on the index esophagogastroduodenoscopy (EGD), as it can be embedded within a background of inflammation in cases of erosive esophagitis in up to 12%. Thus, in such cases, the patient should be treated with a proton-pump inhibitor (PPI) and a repeat EGD to be performed at a latter date, at least at eight weeks, especially when the intention is screening for BE.[43] In addition, when endoscopic lesions are visible in a patient with known HGD, the risk of having EAC with invasion beyond the mucosa is higher than those without.[44] Conventionally, the length of BE lesions is measured by subtracting the distance from the incisors to the squamocolumnar junction from the distance from the incisors to the top of the gastric folds using the regular gastroscope. Even though this method has been demonstrated to be outperformed by gastroscopes marked at 1 cm as opposed to the traditional 5-cm intervals in the accuracy of measurements.[45,46] Another method for measuring the surface area of BE lesion size, designated as quantitative endoscopy (QE), was shown to be a safe and accurate way of following up BE lesions.[47] However, the recently developed and validated Prague C and M criteria for endoscopic diagnosis of BE has excellent landmark recognition of the squamocolumnar junction, gastroesophageal junction, the extent of circumferential columnar lining, and the most proximal extension of the columnar mucosa not accounting for islands of BE[48] and should be used as a standard method for describing BE. Although using methylene blue (MB) can selectively stain intestinal metaplasia, and the intensity of staining does correlate with the histological degree of dysplasia, it was not proven to be superior in the detection of dysplasia compared with the conventional four-quadrant biopsies (4QB).[49–52]

## PATHOLOGICAL FINDINGS

The histological findings on endoscopically obtained biopsies might be one of the most important facets that the management of BE hinges on. In BE, the normal squamous epithelium is substituted with glandular mucosa that is composed of metaplastic columnar cells and goblet cells with their distinct ovoid mucin droplets that can be identified by its blue staining reaction with Alcian blue and by the use of other immunohistochemical stains. Dysplasia, on the other hand, is defined as neoplastic epithelium that remains confined within the basement membrane of the epithelial surface within which it arose. Sampling error is a major concern in patients with BE, which stems from the observation that IM is a mosaic of three distinct epithelial patterns within the columnar lined esophagus and can be unifocal, multifocal, or diffuse.[53] In addition there is poor interobserver reproducibility of pathological grading of biopsies even where a high volume of BE patients are seen.[54] New methods that are used with the aim of assessing the prognosis of BE progressing to HGD or EAC are cytological studies, DNA ploidy analysis with digital image analysis, and fluorescence in situ hybridization. Although it has been demonstrated that the detection rate of IM is related to the number of biopsy obtained,[55] systematic 4QB was found to be more effective than random biopsies.[56] A technique for obtaining biopsies, coined, the “turn and suction” method results in a 56% longer biopsy specimen compared with the traditional method.[57] Also, the use of an angled swingjaw forceps results in larger biopsy samples and a better quality of tissue obtained when compared with conventional forceps.[58]

With the advent of endoscopic ablation procedures for BE, histological regression, in addition to endoscopic regression, has been assessed as an endpoint for response to therapy. Histological regression of IM and dysplasia is seen as soon as one month after the application of ablation procedures as proven by immunohistochemistry, proliferative capacity, and DNA ploidy.[59]

## SCREENING AND SURVEILLANCE

As there is no current evidence that screening the general population for BE has an impact on the mortality from EAC, It has not been recommended at this point of time, and targeting populations at higher risk for BE are only recommended based on expert opinion and should be applied on an individual basis.[2] The value of surveillance programs in patients with BE has been a point of debate.[60,61] It could be argued to be worthwhile given the results of a database-derived study that demonstrated that when an EGD was performed one year prior to the diagnosis of EAC, the patients were diagnosed at an earlier stage and had improved survival rates.[62] Furthermore, a second study found that when a surveillance program was applied in patients with BE, there was a 2% per year cure rate from cancer and a reduction in treatment costs.[23] These programs would have a larger impact if patients at high risk for progression to HGD or EAC could be identified. When considering the initiation of a surveillance program for a patient with BE, it should be an informed decision with the understanding of the risks, benefits, limitations, and willingness to adhere to this process.[2] Sampling of BE should be performed in a 4QB fashion every 2 cm and each segment submitted for pathology separately, so that focused biopsies can be performed if dysplasia is detected. When there is no dysplasia, a second EGD with biopsies should be performed within a year and then every three years. In the case of low-grade dysplasia (LGD), it should be confirmed by a gastrointestinal pathologist, and an EGD should be repeated after six months and then annually till two consecutive EGDs demonstrate no dysplasia. When HGD is detected on flat mucosa, it should be confirmed by an experienced gastrointestinal pathologist, and a repeat EGD performed within three months with 4QBs at 1-cm intervals,[63] because of the risk of concomitant early EAC. If mucosal irregularity is found with HGD, endoscopic mucosal resection (EMR) should be performed and staging of the patient should be attempted because of the high risk of progression to EAC. The therapeutic options such as surgical, endoscopic ablation, and intense surveillance are presented to the patient at this time. Repeated surveillance is recommended after ablative procedures are performed or if dysplasia is lost, as it has been demonstrated that HGD can recur, as well as the everlasting question of buried segments of Barrett's mucosa as glands or islands under the neosquamous epithelium.

## MEDICAL VERSUS SURGICAL APPROACHES

Although an initial study showed that a higher PPI dose resulted in better reduction of gastric pH in the normal population,[64] in patients with BE, regardless of the PPI dose used, the gastric pH was >4 in 80–88% of patients, but the intraesophageal pH remained <4 for >5% of the time.[65] Results from the LOTUS trial (a large multicenter randomized European study), with a three-year follow-up, showed that the esophageal pH was better controlled in patients with BE who underwent laparoscopic antireflux surgery (LARS), compared with patients treated with a PPI, but the symptom outcomes were the same.[66] However, another study found that neither surgical nor medical management of patients with BE altered its natural history,[67] and in a recent meta-analysis by Li *et al*, neither pharmacologic nor surgical antireflux measures achieved complete regression of BE, nor eliminated the risk of EAC.[68] Two retrospective studies demonstrated a reduced risk of dysplasia when patients with BE used PPIs.[69,70]

Complex surgeries involving duodenal diversion procedures in addition to antireflux surgeries[71] have been abandoned. Esophagectomy for early EAC in patients with BE still remains the standard of care, but this is associated with a high morbidity rate, and endoscopic ablative procedures are a viable option especially in high-risk patients.[72,73] Factors that were found to be associated with patients undergoing an esophagectomy as opposed to endoscopic ablation procedures were age ≤60, cancer stage T1sm or greater, and initial consultation performed by a surgeon as opposed to a gastroenterologist.[74] In cases with BE and HGD or early intramucosal adenocarcinoma (IMC), esophagectomy has been demonstrated to have excellent survival outcomes and low mortality rates.[75,76] Also, vagal-sparing esophagectomy has been shown to have a less perioperative morbidity and a shorter hospital stay with less late complications such as weight loss, dumping, and diarrhea compared with transhiatal or en bloc esophagectomy.[77]

### Endoscopic ablation techniques

A large bulk of research in the field of BE has been devoted to the endoscopic prevention of progression of BE to adenocarcinoma or what is called endoprevention.[78] Despite that a number of trials have been conducted for the different modalities for endoablation of BE, they had different endpoints, methodologies, and used different patient populations of BE.[79] The aim of these endoprevention techniques is the regression of Barrett's mucosa to squamous reepithelialization. All of the studies incorporated acid suppressive therapy using either once daily or twice a day PPI. Argon plasma coagulation (APC) has been used to treat HGD and even small EAC lesions in a multicenter RCT. APC achieved regression of BE in 69–97% of patients.[80–83] Similar results were obtained in an earlier study.[84] Some of the complications of this treatment modality are dysphagia, strictures, chest pain, nausea, vomiting, and fever.[81,84,85] As mentioned earlier, there is always a risk of recurrence even after regression of BE. Some of the factors that predict APC failure after initial reepithelialization are persistence of acid reflux and long segments of BE.[86] Photodynamic therapy (PDT) is one of the earliest ablative techniques applied for BE,[87] which utilizes different photosensitizing drugs followed by endoscopic laser light exposure of the segment of BE at a wavelength of 630 nm. The advantage of PDT is its ability to deliver a more targeted therapy and the capacity of giving repeated dosages.[88] A large multicenter RCT demonstrated that PDT decreases the likelihood of high-grade dysplasia (HGD) [absolute risk reduction (ARR) of 38%], cancer (ARR 14%), and a longer time to progression to cancer in patients with BE.[89] Similar results were found in a few earlier studies.[82,85,90] Some of the side effects associated with PDT are cutaneous photosensitivity, odynophagia, and stricture formation.[87] The risk of stricture formation was found to be related to the length of BE, the number of treatment sessions, previous strictures, and the use of EMR prior to PDT.[91,92] Multipolar electrocoagulation (MPEC) was used in a RCT in a repeated fashion every four to eight weeks until endoscopic reversal of BE or up to six sessions, reversal of BE was achieved in 75–88% of patients.[80,81] A meta-analysis demonstrated that endoscopic ablation for BE was capable of achieving endoscopic and histological reversal of BE, and APC appeared to be more effective than PDT, but there was no statistical difference between APC and MPEC. These studies were underpowered to detect a reduction or prevention of progression to adenocarcinoma.[68] EMR has demonstrated good results for the treatment of patients with HGD and intramucosal adenocarcinoma (IMC) and demonstrated eradication of BE in 88% after a median follow-up of 28 months.[93] Both the “inject, suck, and cut” and “band and snare” techniques yield equivalent and adequate depth of histological specimens.[94] An advantage of using EMR in BE is the ability to stage superficial neoplasms when present in tandem with endoscopic ultrasound[95,96] with good interobserver agreement when interpreting the specimens obtained.[97,98] Circumferential balloon-based ablation using radiofrequency energy is a modality that has been proven to be safe and effective for ablation of BE with HGD[99–102] with complete ablation of BE in 90% of patients after a median follow-up of 12 months.[103] Also in another study, complete ablation was achieved in 98% of patients after 2.5 years.[104] When compared with surgery, endoprevention is associated with a higher risk of progression of adenocarcinoma, whereas surgery has a higher cost and results in more frequent minor complications but is curative.[105]

### Nonconventional imaging techniques

Because of the ease of accessibility of the esophagus, it has been an area of intense research for different imaging techniques. Narrow-band imaging (NBI) is based on using interference filters for the illumination of the mucosa with narrowed blue and green bands of the light spectrum in combination with magnifying endoscopy.[106] It better visualizes and discriminates between the mucosal glandular structures and vascular architecture, when compared with standard resolution white light endoscopy. NBI is better in detecting HGD with a less number of biopsies,[107,108] and the sensitivity of NBI for HGD in BE was found to be 86%.[109] A simplified classification of mucosal morphology has been validated and found to have a good correlation with histological diagnosis (88%) and good reproducibility regardless of the expertise with NBI use.[110] Confocal laser microscopy (CLM) is mainly used to examine a small segment of the mucosa as opposed to other modalities where the whole esophagus can be examined. A large prospective German study conducted in two phases to establish the criteria for the diagnosis of neoplasia in cases of normal macroscopic appearing mucosa in patients with BE showed that compared with standard high resolution endoscopy (HRE), CLM had a higher negative predictive value (98.8%) for neoplasia in BE but had a poor positive predictive value (44%), these results need further validation on a larger scale.[111] A number of studies have proven that capsule endoscopy is inadequate in the investigation of patients with suspected BE or esophageal disease in general.[112,113] Magnification chromoendoscopy is able to detect IM in BE.[114] The sensitivity of HRE with indigo carmine chromoendoscopy for HGD in BE is in the range of 93%,[109] whereas using 0.05% crystal violet had a sensitivity of 89% and a specificity of 86%.[115] Computed virtual chromoendoscopy (CVC) is a new imaging modality that enhances the mucosal surface using contrast and delineates the vascular pattern, it has been demonstrated to be as sensitive as conventional chromoendoscopy and with a positive predictive value of only 39%.[116] Another method utilizes autofluorescence endoscopy (AFE) for targeted biopsies in the surveillance of BE. In a multicenter study, AFE was shown to improve the diagnostic yield for neoplasia in comparison with 4QB but was not suitable for replacing the standard 4QB method.[117] Also light-induced fluorescence endoscopy did not enhance the detection of HGD.[118] Although an initial study suggested that magnification endoscopy was superior to standard 4QB in detecting HGD,[119] a second prospective randomized trial showed that there was no added advantage in using enhanced magnification endoscopy in the surveillance of BE.[120] A possible explanation of these apparent discordances in the results is the high interobserver variability and mismatch between cardiac mucosa and nondysplastic Barrett's mucosa.[121] Raman spectroscopy is based on the principle of inelastic scattering of monochromatic light usually from a laser; it has a promising role in being an adjunct in the surveillance of patients with BE but is still under evaluation, as is optical coherence tomography.[122]

## CURRENT STATUS OF PRACTICE

A number of guidelines have been issued from different societies regarding the diagnosis and management of patients with BE.[123–125] However, even with the widespread dissemination of these guidelines, our practice in the management of BE is far from perfect and studies have demonstrated wide variations between gastroenterologist practices in the management of BE and the guidelines issued.[126–131]

## FUTURE TRENDS

The hope is that we can better characterize factors that promote the development of BE and its progression to HGD and EAC and evaluate different biomarkers that can identify this subset of patients. Also there have been efforts toward developing a vaccine for esophageal cancer, with promising results.[132,133] Also the efficacy of surveillance programs requires further investigation in addition to the establishment of specialized BE clinics in high prevalence areas that would have a more structured approach to this patient population.[134] The issue of endoscopy units being overcrowded with other procedures is a reality that gastroenterologists deal with on a daily basis. One approach would be using an office-based unsedated small-caliber endoscopic approach, although the biopsy samples obtained would be smaller than that of conventional endoscopy, but the accuracy of this approach in BE screening is acceptable.[135,136]
